# Human Bocavirus and Gastroenteritis

**DOI:** 10.3201/eid1310.070436

**Published:** 2007-10

**Authors:** Oliver Schildgen, Andreas Müller, Arne Simon

**Affiliations:** * University of Bonn, Bonn, Germany

**Keywords:** bocavirus, gastroenteritis, letter

**To the Editor:** We read with great interest the recent study by Vicente and colleagues, who suspect the human bocavirus (HBoV), a newly detected parvovirus initially described as a respiratory pathogen, to be a possible causative agent of gastroenteritis in children (*1*). These researchers investigated the presence of HBoV DNA in 527 stool samples from ambulatory patients (<36 months of age) with unrelated respiratory symptoms. Of these stool samples, 48 (9.1%) were positive for HBoV DNA. Other enteric pathogens were found in 58% of all HBoV-positive fecal samples.

A close taxonomic relationship exists between HBoV and bovine parvovirus, an animal virus capable of causing gastrointestinal symptoms in cattle (*2*). Taking into account the assumed high tenacity of this parvovirus against environmental factors and hospital-grade disinfectants (*3*,*4*), we believe the possibility of fecal-oral transmission, in addition to transmission via respiratory droplets, has to be considered in interpreting the observations of Vicente et al. (*1*).

Gastroenteric symptoms have been described in up to 25% of all patients with respiratory HBoV infections (*5*–*7*). Although these observations suggest that HBoV may contribute to gastroenteritis or even be a causative agent, further studies are needed. Such studies should include control groups of asymptomatic patients and should test stool samples for HBoV. The correlation between detection of HBoV and clinical symptoms of gastroenteritis needs further confirmation in animal models, which are still not available. Nevertheless, the study by Vicente et al. did not clarify the extent of respiratory symptoms in patients with HBoV-positive stool samples. Taking into account the nature of parvovirus particles, we believe the virus likely passed through the gastrointestinal tract, as patients frequently swallow virus-containing sputum or nasal secretions. Thus, the observation that HBoV is an enteric pathogen should be considered a preliminary finding. Finally, we suggest that the role of HBoV should be investigated through histologic examination of mucosa biopsy specimens (e.g., from patients with chronic gastrointestinal diseases) to confirm pathogenicity.
