# Supplementary material for: Human Bocavirus and Gastroenteritis
Source: Emerg Infect Dis. 2007 Oct;13(10):1620–1. doi: 10.3201/eid1310.070436 (PMC2851507; doi:10.3201/eid1310.070436)
Supplement: Related texts

# Letter

**DOI:** 10.3201/eid1310.070934

**Published:** 2007-10

**Authors:** Diego Vicente, Gustavo Cilla, Milagrosa Montes, Eduardo G. Pérez-Yarza, Emilio Pérez-Trallero

**Affiliations:** * Hospital Donostia, San Sebastián, Spain; † CIBER Enfermedades Respiratorias, San Sebastian, Spain; ‡ Universidad del País Vasco, San Sebastián, Spain

## In Response

We thank Schildgen et al. (*1*) for their comments on our study, with which we basically agree. Our main findings were the possibility of fecal–oral transmission of the recently discovered human bocavirus (HBoV) and the high frequency with which it has been detected in the feces of infants and young children with gastroenteritis (*2*). For us, this latter finding suggested that HBoV is an enteric pathogen, although we cannot rule out the possibility that the fecal elimination was unrelated to the diarrhea in these patients. The possible pathogenic role of this virus in acute respiratory infections has also been questioned; consequently, several types of studies have been proposed to obtain evidence of pathogenicity and proof of disease causation (*3*,*4*).

Schildgen et al. suggest the possibility that HBoV could be detectable in feces after patients swallow respiratory tract secretions. Most of the children with gastroenteritis in our study showed no signs of respiratory infection. Swallowing respiratory tract secretions when one does not have respiratory inflammation would probably produce a quantity of virus too small to be detected in feces in the high frequency with which it was detected in our study. Therefore, we believe that the fecal elimination was more likely produced by HBoV replication in the intestinal mucosa. The studies proposed by Schildgen et al. would undoubtedly help elucidate this issue.

## References

[1] Schildgen O, Müller A, Simon A. Human bocavirus and gastroenteritis [letter]. Emerg Infect Dis. 2007;13:1620.1825802610.3201/eid1310.070436PMC2851507

[2] Vicente D, Cilla G, Montes M, Pérez-Yarza EG, Pérez-Trallero E. Human bocavirus, a respiratory and enteric virus. Emerg Infect Dis. 2007;13:636–7. 10.3201/eid1304.06150117553287PMC2725986

[3] McIntosh K. Human bocavirus: developing evidence for pathogenicity. J Infect Dis. 2006;194:1197–9. 10.1086/50822817041844PMC7109811

[4] Allander T, Jartti T, Gupta S, Niesters HGM, Lehtinen P, Osterback R, Human bocavirus and acute wheezing in children. Clin Infect Dis. 2007;44:904–10. 10.1086/51219617342639PMC7107819
